# Dietary Intakes of Elite Male Professional Rugby Union Players in Catered and Non-Catered Environments

**DOI:** 10.3390/ijerph192316242

**Published:** 2022-12-04

**Authors:** Logan Posthumus, Matthew Driller, Katrina Darry, Paul Winwood, Ian Rollo, Nicholas Gill

**Affiliations:** 1 Faculty of Health, Education and Environment, Toi Ohomai Institute of Technology, Tauranga 3112, New Zealand; 2 Te Huataki Waiora School of Health, The University of Waikato, Hamilton 3216, New Zealand; 3 New Zealand Rugby, Wellington 6011, New Zealand; 4 School of Allied Health, Human Services and Sport, Sport and Exercise Science, La Trobe University, Melbourne 3086, Australia; 5 Department of Sport and Recreation, Sports Performance Research Institute New Zealand, Auckland University of Technology, Auckland 0627, New Zealand; 6 Gatorade Sports Science Institute, PepsiCo Life Sciences, Global R&D, Leicestershire LE4 1ET, UK

**Keywords:** dietary analysis, body composition, macronutrients, energy intake, team-sport

## Abstract

In professional rugby union, it is common for players to switch between catered and non-catered dietary environments throughout a season. However, little is known about the difference in dietary intake between these two settings. Twelve elite male professional rugby union players (28.3 ± 2.9 y, 188.9 ± 9.5 cm, 104.1 ± 13.3 kg) from the New Zealand Super Rugby Championship completed seven-day photographic food diaries with two-way communication during two seven-day competition weeks in both catered and non-catered environments. While no significant differences were observed in relative carbohydrate intake, mean seven-day absolute energy intakes (5210 ± 674 vs. 4341 ± 654 kcal·day^−1^), relative protein (2.8 ± 0.3 vs. 2.3 ± 0.3 g·kgBM·day^−1^) and relative fat (2.1 ± 0.3 vs. 1.5 ± 0.3 g·kgBM·day^−1^) intakes were significantly higher in the catered compared to the non-catered environment (respectively) among forwards (*n* = 6). Backs (*n* = 6) presented non-significantly higher energy and macronutrient intakes within a catered compared to a non-catered environment. More similar dietary intakes were observed among backs regardless of the catering environment. Forwards may require more support and/or attention when transitioning between catered and non-catered environments to ensure that recommended dietary intakes are being achieved.

## 1. Introduction

Rugby Union (RU) is a high-intensity collision-based team sport contested all over the world at the amateur, semi-professional, and professional levels. During the in-season period, professional RU players are exposed to substantial training and game demands [1,2]. Within a competition week, players can cover total distances of ~23 km and present relatively high internal training loads [1]. Throughout an 80 min game (comprising two 40 min halves), players cover total distances of ~6 km [1] and are involved in numerous collisions [3]. During both training and game-play, players are exposed to substantial exercise and impact-induced muscle damage [4,5] and expend considerable amounts of energy during a competition week [6,7,8]. Due to the collisions experienced during a game, players may have additional energy requirements, evident through elevated resting metabolic rates following game-play [9,10,11,12].

Currently, the nutritional practices of professional RU players have reported common trends regarding dietary intake. Players consume a relatively low carbohydrate (~3.5 g·kg of Body Mass [BM] per day^−1^), high protein (>2.0 g·kgBM·day^−1^) and moderate-high fat diet (>1.4 g·kgBM·day^−1^) [1,6,10,13,14] compared to nutritional recommendations for team-sport athletes (carbohydrate = 5–8 g·kgBM·day^−1^; protein = 1.7–2.2 g·kgBM·day^−1^; fat = 30–50% of total energy intake) [15,16]. It is recommended that team-sport athletes consume an adequate energy and macronutrient intake in order to offset energy expenditure, increase glycogen stores and repair body tissues [15]. To this end, sufficient dietary intake plays an integral role in player health and is also required to drive positive adaptations from training and game demands [17].

Studies have examined dietary intakes in-season among professional RU players [1,6] and various team-sport athletes [15]. However, to our knowledge, no studies have examined the effects of a catered versus non-catered environment on dietary intakes among elite male professional RU players. It is important to understand the impact of the food environment on dietary intakes among professional RU players since transitioning between catered and non-catered environments is common [18]. Depending on the rugby club and its resources, professional players may be required to prepare their own meals when playing home games, but may be catered for when playing away games due to staying in hotels when travelling. Furthermore, players may be in a catered (hotel) environment routinely or for extended periods of time. Hotel catering represents a significantly altered dietary environment compared to home catering, with buffet options for main meals and a team room with various snack options available at all times.

Players in a catered (hotel or fully catered team facility) environment may better achieve dietary intake recommendations for team-sports [15] compared to a non-catered (preparing own meals) environment. This is because, in a catered environment, players need only focus on appropriate food selection and portion sizes, as long as suitable food options are provided [19]. Conversely, in a non-catered environment players must also focus on food shopping, budgeting, family requirements, and cooking skills [19], which are common barriers to the implementation of nutrition plans [20,21]. Understanding the ad libitum energy intake of RU players in catered and non-catered environments would provide valuable information on how the environment influences dietary intake. These insights may also be valuable for academy and semi-professional rugby players who transition into the professional game, where the exposure to catered environments is increased.

Therefore, the aim of this study was to examine the dietary intakes of elite professional RU players in catered and non-catered environments during two seven-day competition weeks. It was hypothesised that players would consume significantly greater energy and macronutrient intakes in a catered compared to a non-catered environment due to the greater availability of and access to food.

## 2. Materials and Methods

### 2.1. Study Design

A comparative repeated-measures design was utilised to assess the in-season dietary intakes of twelve elite professional RU players during two seven-day competition weeks in both catered and non-catered environments. Seven-day photographic food diaries were used to capture the dietary intakes during the competition weeks in both catered and non-catered environments. The same forwards and backs took part in the non-catered (FNC and BNC, respectively) environment first, followed by the catered (FC and BC, respectively) environment two weeks later. All players were experienced and familiar with recording dietary intakes and had experience in both catered and non-catered environments prior to the commencement of the study, reducing the risk of learning and/or order effects. Dietary intake across the competition week was conveyed as days away from game day (GD [GD-5, GD-4, GD-3, GD-2, GD-1, GD, GD + 1]) as previously presented [1,6,8].

All players within this study competed on GD in both the catered and non-catered competition weeks, with the game starting at ~1900 h. The catered environment involved a hotel which provided breakfast, lunch, and dinner. Snacks consisting of a range of food choices, were also available ad libitum via a team room whenever required. The non-catered environment involved players’ personal homes, where the players were required to cater for themselves at breakfast, lunch, dinner and snacks. In both conditions, players were provided with pre- and post-training meals optimised for fuelling and recovery around training. On GD, all players were provided with pre- and post-game meals which were similar to those previously reported [1]. Players were provided with the same supplements in both conditions which consisted of multi-vitamins, fish oils, protein powders, pre-workouts and sports drinks.

All players were provided with nutrition education in both catered and non-catered environments, focusing on the intake of macronutrients to meet energy requirements for the maintenance of body mass and fuelling for performance. The food in the catered environment was designed and selected by the team’s sports nutritionist. A buffet-style system was provided at breakfast, lunch and dinner for players to serve themselves. Catering followed best practice principles, both regarding hand sanitisation prior to handling plates and cutlery and the provision of information cards labelling foods [19]. Vegetable/salads and carbohydrate options were available first in the buffet selections alongside healthy nuts, seeds, and oils in order to promote these foods prior to protein-based options [19,22,23]. Players were encouraged to explore buffet choices before serving up in order to pre-select food choices and avoid overloading [19].

### 2.2. Participants

Twelve elite male professional RU players from the New Zealand Super Rugby Championship participated in the current study. Players were categorised by their primary playing position, which comprised six forwards (F; Hookers = 1, Locks = 3, Loose = 2) and six backs (B; Half-Back/Scrum-Half = 1, First-Five/Fly Half = 1, Mid-Field = 2, Outside = 2). Years of professional experience (quantified by when players were first selected for a professional rugby team) for forwards and backs were 9.0 ± 2.8 and 9.2 ± 2.7 y, respectively. One additional forward (prop) and one additional back (outside) were initially recruited for this study but were withdrawn due to team selection changes. For players to be included in this study, they had to be selected and compete on GD in both competition weeks/environments. Informed consent was obtained from all participants involved in the study. The study was conducted according to the guidelines of the University of Waikato and approved by the University of Waikato Human Research Ethics Committee (HREC 2019#04).

### 2.3. Anthropometrics and Body Composition

Player’s stature and body mass (BM) were collected at the start of the competition week (GD-5). Upon waking with bladder voided, BM was assessed using electronic scales (SECA, Birmingham, UK) configured to 0.1 kg accuracy. Stature was then immediately assessed using a stadiometer (SECA, Birmingham, UK) configured to 0.5 cm accuracy. Sum of eight site skinfolds (triceps, subscapular, biceps, iliac crest, supraspinale, abdominal, mid-thigh, and medial calf) were collected using Harpenden callipers (British Indicators, Hertfordshire, UK), configured to 0.1 mm accuracy by a level 1 International Society for the Advancement of Kinanthropometry (ISAK) accredited anthropometrist following the same methods as previously described [24]. Total fat-free mass (FFM) and body fat percentage (Fat %) were collected one week prior to the commencement of the study using a fan-beam Dual-energy X-ray Absorptiometry (DXA) scanner (Hologic Discovery A, Hologic, Bedford, MA, USA) using the same methods previously described [24].

### 2.4. Training and Game Load

Quantification of internal game load, as well as field and gym training loads, were expressed using session rate of perceived exertion (sRPE) to provide arbitrary units (AU) using methods described previously [1,8]. Quantification of external game load and field training load were expressed using total distance values, collected via 10 Hz global positioning systems (GPS) units (VX Sport, Wellington, NZ) and software (VX Sport, Wellington, NZ), following methods previously described [1,25]. These internal and external training and game loads were collected to provide context alongside the seven-day dietary intakes collected (Table 1).

### 2.5. Dietary Intake Assessment and Analysis

A seven-day remote photographic food diary/Snap-N-Send method was utilised to examine dietary intakes using methods previously described [1,26,27]. Players used the smartphone application ‘WhatsApp’ to photograph what they consumed at every eating/drinking occasion and provided a brief description of any foods, brand names, cooking methods, or items difficult to quantify and/or identify for the analyser. All players received appropriate training to ensure they were proficient at capturing this information. Two-way communication between player and the teams sports nutritionist were implemented to ensure that all details were correct [1]. These photographs and descriptions were then analysed and manually entered into a dietary analysis software (FoodWorks 10 Professional, Xyris, Australia) by a single registered sports dietitian.

### 2.6. Statistical Analyses

All statistical tests were analysed using the Statistical Package for the Social Sciences (SPSS, v28, IBM, New York, NY, USA). All data were checked for normality using the Shapiro–Wilk test. Differences between catered and non-catered environments within forwards and backs for mean seven-day energy and macronutrient intakes were assessed using paired *t* tests. A two-way repeated measures analysis of variance was utilised to determine differences between absolute energy and relative macronutrient intakes across time for forwards (FC vs. FNC) and backs (BC vs. BNC) in catered and non-catered environments. An alpha value of *p* ≤ 0.05 was utilised for all tests. Effect sizes were calculated using the Cohens *d* method with the following thresholds: *d* = *trivial* < 0.19, *small* 0.20–0.49, *medium* 0.50–0.79, and *large* > 0.80 [28]. All data are expressed as mean ± SD.

## 3. Results

### 3.1. Demographics

Demographics and body composition can be observed in Table 2. Forwards demonstrated significantly greater stature (*p* = < 0.01; *d* = 2.13), BM (*p* = < 0.01; *d* = 3.02), FFM (*p* = < 0.01; *d* = 2.87), and Fat % (*p* = 0.02; *d* = 1.65) compared to backs.

### 3.2. Seven-Day Dietary Intakes

Mean seven-day energy and macronutrient intakes can be observed in Table 3. Significantly greater absolute (*p* = < 0.01; *d* = 1.86), relative to BM (*p* = < 0.01; *d* = 1.84) and relative to FFM (*p* = < 0.01; *d* = 1.85) energy intakes were observed for FC compared to FNC. Significantly greater absolute (*p* = < 0.01; *d* = 2.29–2.04), relative to BM (*p* = < 0.01; *d* = 2.07–2.10), and relative to FFM (*p* = < 0.01; *d* = 2.37–2.17) protein and fat intakes were observed for FC compared FNC. No significant differences were observed in energy and macronutrient intakes among BC and BNC.

All forwards reported higher mean seven-day energy intakes in the catered compared to non-catered environment. Meanwhile, two out of the six backs consumed less in a catered (3922 ± 648 and 3335 ± 731 kcal·day^−1^) compared to a non-catered environment (4191 ± 697 and 3490 ± 563 kcal·day^−1^). No significant differences were observed in the mean number of meals consumed per day between environments among forwards and backs.

### 3.3. Daily Absolute Energy and Relative Macornutrient Intakes

**Figure 1:**
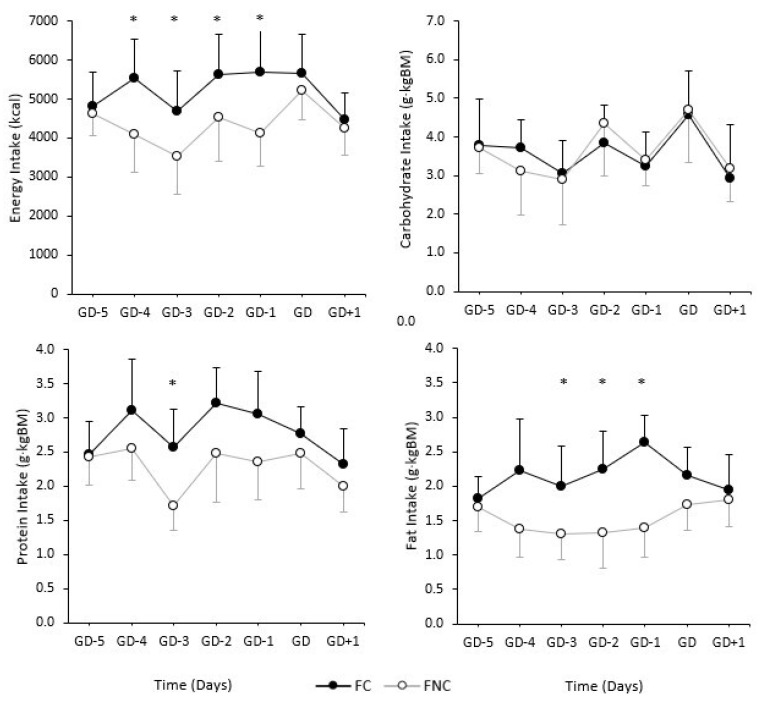
Daily absolute energy intake and relative macronutrient intake during competition weeks (GD-5, GD-4, GD-3, GD-2, GD-1, GD, GD + 1) among forwards within a catered (FC) and non-catered (FNC) environment. Error bars represent standard deviation. * Indicates a significant difference between FC and FNC.

**Figure 2:**
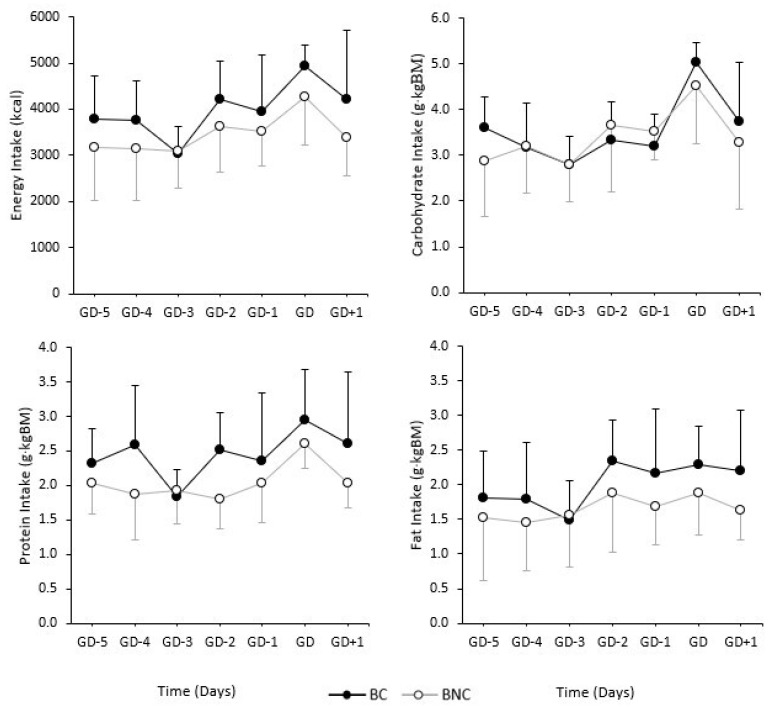
Daily absolute energy intake and relative macronutrient intake during competition weeks (GD-5, GD-4, GD-3, GD-2, GD-1, GD, GD+1) among backs within a catered (BC) and non-catered (BNC) environment. Error bars represent standard deviation.

Mean daily averages for absolute energy intake and relative macronutrient intake can be observed in Figure 1 and Figure 2 for forwards and backs, respectively. Significantly greater absolute energy intakes were observed on GD-4 (*p* = 0.03; *d* = 1.30), GD-3 (*p* = 0.03; *d* = 1.29), GD-2 (*p* = 0.04; *d* = 1.05), and GD-1 (*p* = 0.04; *d* = 1.08) for FC compared to FNC. Significantly greater relative protein intakes on GD-3 (*p* = 0.01; *d* = 1.59) and significantly greater relative fat intake on GD-3 (*p* = 0.03; *d* = 1.24), GD-2 (*p* = 0.02; *d* = 1.43), and GD-1 (*p* = < 0.01; *d* = 2.05) were observed for FC compared to FNC. No significant differences were observed between BC and BNC. 

## 4. Discussion

This study was the first to compare the dietary intakes of elite male professional RU players during competition weeks in both catered and non-catered environments. Forwards in a catered environment consumed significantly greater mean seven-day energy, protein, and fat intakes compared to a non-catered environment. Additionally, significant differences were present among forwards for daily absolute energy and relative protein and fat intakes between catered and non-catered environments. Backs demonstrated no significant differences in mean seven-day or daily energy and macronutrient intakes between environments. The mean seven-day energy intakes observed within catered environments for both forwards and backs were higher than previously reported among southern [1] and northern [6] hemisphere players during an in-season period. However, the non-catered energy intakes among forwards and backs were lower than previously reported in the southern hemisphere [1], but higher than those for northern hemisphere [6] players during the in-season.

Mean seven-day relative carbohydrate intakes were alike for both forwards and backs in catered and non-catered environments, which was similar to previously reported intakes [1,6]. Relative carbohydrate intakes were slightly higher in catered forwards and backs compared to non-catered, however these differences were negligible. When expressed as a percentage of total calorie intake, carbohydrates were 6% and 3% higher in non-catered environments for both forwards and backs, respectively. This may be due to carbohydrate-rich foods being more cost-effective and convenient to fill the plate with when self-catering at home compared to more expensive, labour-intensive protein-rich foods [20,21]. Whereas, multiple protein options and larger quantities are often available in catered compared to non-catered environments [19,23].

Regardless of catering or geographic location, ~3.5 g·kgBM·day^−1^ is the average carbohydrate intake reported for professional RU players [1,2,6,10,13,14], which represents the lower value in the range recommended for team-sport athletes [15]. However, average carbohydrate intakes were higher (4–5g·kgBM) on intense training days (particularly GD-2) for forwards in both environments and on GD for both forwards and backs in both environments, as seen in previous studies [1,6,10]. These data demonstrate evidence of carbohydrate intake periodisation based on training load [1,29,30]. Additionally, though carbohydrate intakes were relatively low across the competition weeks, mean seven-day dietary fibre intakes among forwards and backs were high compared to recommended daily intakes (> 30 g·day^−1^) and other team-sport athletes [31,32]. This information suggests good quality food sources are being consumed in both environments, which may promote a range of health benefits while reducing the risk of nutrition-related lifestyle diseases [33].

Mean seven-day relative protein intakes were higher in the catered compared to the non-catered environment among forwards and backs. These protein intakes align with current reported ranges among professional RU players during the in-season period (2.0–2.7 g·kgBM·day^−1^) [1,6,10,34], with catered forwards and backs being towards the higher end of this range. Protein intakes may be higher in catered environments due to greater variety and multiple options of different protein sources being available to players [19]. These higher protein intakes are substantial given that an additional 0.4–0.5 g·kgBM can have positive effects in promoting muscle protein synthesis among certain populations [16,35]. Higher protein intakes may be important for supporting growth and recovery in professional RU players given the considerable exercise and impact-induced muscle damage experienced from gym and field training sessions and gameplay [3,4,5,9,10,11,12].

Mean seven-day fat intakes were higher for catered compared to non-catered forwards and backs. These fat intakes within a catered environment were substantially higher than previously reported in-season among northern (1.4 ± 0.3 g·kgBM·day^−1^) [6,10] and southern hemisphere (1.8 ± 0.4 g·kgBM·day^−1^) players [1], but similar to those of players with a weight gain goal in a southern hemisphere pre-season study (2.0 ± 0.4 g·kgBM·day^−1^) [14]. Fat intakes within a non-catered environment were more similar to those of northern hemisphere players [6]. These higher fat intakes from the catered environments may derive from greater protein intake and players selecting a greater amount of healthy fat options such as; olive oil, avocado, nuts and seeds at meal times. A potential positive regarding these increased fat intakes is that it may help some players meet energy requirements without the feeling of having to consume too much food. For example, with similar carbohydrate intakes between catered and non-catered conditions, the greater mean energy intakes observed in a catered environment are mainly due to higher protein and fat intakes, with fat contributing substantially more absolute energy difference between FC and FNC (549 kcal) and BC and BNC (306 kcal).

Over the course of the competition weeks there were no significant differences in daily energy and macronutrient intakes between environments for backs. Whereas, absolute energy intake was significantly higher for catered compared to non-catered forwards on GD-4, GD-3, GD-2, and GD-1. Relative protein intake was significantly higher on GD-3 (rest and recovery day) in catered compared to non-catered forwards. Meanwhile, relative fat intake was significantly higher on GD-3, GD-2, and GD-1 in catered compared to non-catered forwards. This would indicate that backs have more similar dietary intakes regardless of the food environment, while forwards may require more support or attention when changing between catered and non-catered environments to ensure recommended and consistent dietary intakes are being achieved.

All together, these observations suggest that even when food is catered for, players still consume a similar carbohydrate intake to previously reported data [15]. To this end, food selection and preparation for carbohydrate does not seem to be an issue between environments, making it a matter of choice. In contrast, players consumed more protein and fat in the catered environment compared to non-catered. These higher protein and fat intakes observed within catered compared to non-catered environments, may be due to multiple protein and fat sources being available [19]. This could potentially be an area to help increase calorie intake (once carbohydrate and protein intake are sufficient first and foremost) in non-catered environments to try better meet the energy demands of rugby players, who have shown typically high energy expenditures [7,8,14,36]. However, the contrary must also be considered in that, players who need to reduce calorie intake should be guided carefully or the environment may need to be modified further for these players.

A limitation of the current study was that exercise energy expenditure was not collected, and therefore energy availability could not be determined. Male athletes are recommended to achieve energy intakes > 40 kcal·kgFFM·day^−1^ after exercise energy expenditure has been taken into account (dietary energy intake (kcal)—exercise energy expenditure (kcal)/FFM (kg)) to optimise physiological functions [37,38]. Our results suggest that some players may be at risk of low energy availability. This is due to our reported measures only being ~50–55 kcal·kgFFM·day^−1^ in catered players and ~40–45 kcal·kgFFM·day^−1^ in non-catered players, prior to factoring in exercise energy expenditure. In particular, forwards and backs within non-catered environments may be at greater risk, however those in catered environments also need to be diligent with their dietary intake strategies. Measuring energy expenditure from exercise is highly recommended in these groups to provide a greater understanding of energy availability.

Other limitations within this study were; granted distance and training load were very similar between environments, there may always be certain activities performed in training that cannot be accounted for or replicated across training weeks that may influence a player’s energy intake, such as specific skills, drills and game plans required in a competition week when preparing for different opposition. However, all efforts were made to ensure training activity was controlled and replicable as best as possible. Lastly, another limitation that is present among dietary analysis studies, is the likelihood of misreporting by players [39] and/or the analyst when assessing photographic food diaries [26,40,41]. Once again, all efforts were made to reduce error in the reporting of dietary intakes within this study.

## 5. Conclusions

Forwards in a catered environment demonstrated significantly greater absolute and relative energy intakes and significantly greater absolute and relative protein and fat intakes compared to a non-catered environment. Conversely, no significant differences were observed among backs in catered and non-catered environments. Significant differences in daily absolute energy intakes and relative protein and fat intakes were observed among forwards between catered and non-catered environments, but no significant differences were observed among backs between environments. Forwards and backs in both catered and non-catered environments achieved team-sport nutritional recommendations for protein and fat intakes, however carbohydrate intake was still on the lower end of recommendations regardless of environment. For teams that have players exposed to both catered and non-catered environments during a season, it may be important to compare dietary intakes within each environment to optimise and align nutritional habits in order to meet appropriate nutritional requirements. Future studies could explore the nutritional intakes of professional RU players when competing away from home, with significant travel compared to regular home games alongside measures of exercise energy expenditure. Studies could also compare macronutrient distribution and food product differences across meals throughout the day between different environments.

## Figures and Tables

**Table 1:** Overview of the seven-day competition week in catered and non-catered environments.

	GD-5	GD-4	GD-3	GD-2	GD-1	GD	GD + 1	Total
**All Players**			Rest and Recovery				Rest and Recovery	
Intensity	Low	High		High	Low	High		
Field Sessions	1	1		2	1	0		5
Gym Sessions	1	1		1	0	0		3
**Distance (km)**								
FC	2.7 ± 1.2	5.7 ± 0.9		4.2 ± 1.0	3.0 ± 1.1	5.1 ± 0.7		20.6 ± 3.9
FNC	2.6 ± 0.9	5.7 ± 0.7		4.4 ± 0.5	2.5 ± 0.7	5.3 ± 0.6		20.4 ± 2.5
BC	3.7 ± 1.0	7.7 ± 1.6		5.4 ± 1.0	3.8 ± 0.7	6.4 ± 0.6		26.9 ± 4.3
BNC	3.8 ± 0.8	7.6 ± 1.3		5.3 ± 0.9	3.7 ± 0.8	6.3 ± 1.1		26.7 ± 4.1
**sRPE (AU)**								
FC	719 ± 87	1258 ± 72		1368 ± 94	243 ± 45	688 ± 95		4276 ± 132
FNC	840 ± 138	1281 ± 163		1365 ± 131	229 ± 43	655 ± 55		4369 ± 293
BC	767 ± 106	1270 ± 100		1375 ± 91	239 ± 39	777 ± 57		4427 ± 205
BNC	781 ± 138	1349 ± 129		1347 ± 74	242 ± 46	743 ± 89		4463 ± 206

Mean ± Standard Deviation. FC = forwards catered, FNC = forwards non-catered, BC = backs catered, BNC = backs non-catered, sRPE = session rate of perceived exertion, AU = arbitrary unit. Low intensity = mean day RPE < 6, high intensity = mean day RPE > 6. No significant differences were present for distance or training load between catered and non-catered environments.

**Table 2:** Player demographics and body composition.

Demographics	All Players (*n* = 12)	Forwards (*n* = 6)	Backs (*n* = 6)
Age (y)	28.3 ± 2.9	28.2 ± 2.9	28.5 ± 3.2
Professional Experience (y)	9.1 ± 2.6	9.0 ± 2.8	9.2 ± 2.7
Stature (cm)	188.9 ± 9.5	195.8 ± 7.3 *	182.0 ± 5.6
Body Mass (kg)	104.1 ± 13.3	115.0 ± 6.8 *	93.2 ± 7.6
Fat-Free Mass (kg)	88.2 ± 10.3	96.5 ± 5.4 *	79.8 ± 6.2
Fat % (%)	15.2 ± 1.3	16.1 ± 1.4 *	14.4 ± 0.6
Skinfolds 8-site (mm)	64.2 ± 11.9	69.9 ± 14.8	58.4 ± 3.5

Mean ± Standard Deviation. * Indicates a significant difference between forwards and backs.

**Table 3:** Mean seven-day dietary intakes among catered and non-catered forwards and backs.

Dietary Intake		Forwards (*n* = 6)		Backs (*n* = 6)	
		Catered (*n* = 6)	Non-Catered (*n* = 6)	Catered (*n* = 6)	Non-Catered (*n* = 6)
Energy	kcal·day^−1^	5210 ± 674 *	4341 ± 654	3952 ± 765	3445 ± 610
	kcal·kgBM·d^−1^	45.8 ± 7.2 *	38.2 ± 6.6	42.8 ± 8.4	37.6 ± 5.9
	Kcal·kgFFM·d^−1^	54.2 ± 8.2 *	45.1 ± 7.6	49.6 ± 9.5	43.1 ± 7.1
CHO	g·d^−1^	408 ± 85	411 ± 89	328 ± 65	317 ± 75
	g·kgBM·d^−1^	3.6 ± 0.9	3.6 ± 0.8	3.5 ± 0.6	3.4 ± 0.7
	g·kgFFM·d^−1^	4.2 ± 1.0	4.3 ± 1.0	4.1 ± 0.7	3.9 ± 0.8
	% TEI	34 ± 4	40 ± 4	37 ± 5	40 ± 8
Protein	g·d^−1^	318 ± 33 *	260 ± 29	223 ± 46	188 ± 11
	g·kgBM·d^−1^	2.8 ± 0.3 *	2.3 ± 0.3	2.4 ± 0.5	2.0 ± 0.1
	g·kgFFM·d^−1^	3.3 ± 0.4 *	2.7 ± 0.3	2.8 ± 0.6	2.4 ± 0.2
	% TEI	25 ± 2	25 ± 2	23 ± 4	23 ± 3
Fat	g·d^−1^	244 ± 34 *	183 ± 47	183 ± 47	149 ± 50
	g·kgBM·d^−1^	2.1 ± 0.3 *	1.5 ± 0.3	2.0 ± 0.5	1.6 ± 0.6
	g·kgFFM·d^−1^	2.5 ± 0.4 *	1.8 ± 0.3	2.3 ± 0.6	1.9 ± 0.6
	% TEI	41 ± 3	35 ± 2	40 ± 5	37 ± 8
Fibre	g·d^−1^	52.2 ± 9.9	48.4 ± 11.7	44.9 ± 11.6	41.3 ± 11.4
Meal #	meals·d^−1^	5.2 ± 0.6	5.1 ± 0.8	4.9 ± 0.3	4.6 ± 0.7

Mean ± Standard Deviation. BM = body mass, CHO = carbohydrate, FFM = fat-free mass, % TEI = percentage of total energy intake, Meal # = number of meals per day. * Indicates a significant difference between forwards catered and non-catered.

## Data Availability

The data presented in this study are available on request from the corresponding author and the permission of all parties involved in the study. The data are not publicly available due to privacy.
